# Supplementary material for: Mouse embryo geometry drives formation of robust signaling gradients through receptor localization
Source: Nat Commun. 2019 Oct 4;10:4516. doi: 10.1038/s41467-019-12533-7 (PMC6778081; doi:10.1038/s41467-019-12533-7)
Supplement: Supplementary file 1 — Supplementary Information [file 41467_2019_12533_MOESM1_ESM.pdf]

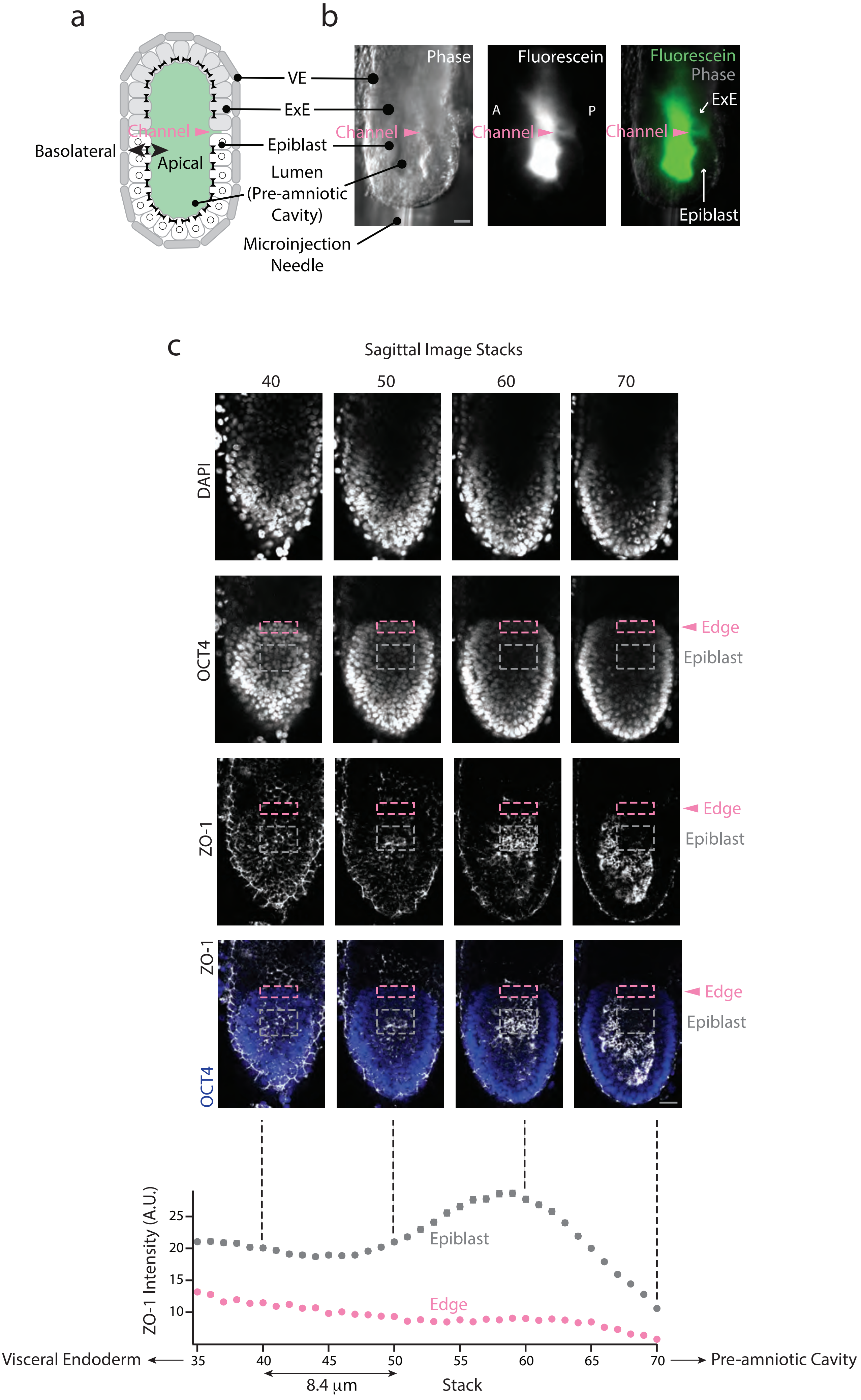

Supplementary Figure 1

Supplementary Fig. 1 | An extracellular channel is present between the epiblast and the extraembryonic ectoderm. a, Illustration of pre-gastrulation mouse embryo, with the epiblast (white) and extraembryonic ectoderm (ExE, light gray) together enclosing the pre-amniotic cavity. Apical membranes of epiblast cells face the pre-amniotic cavity whereas basolateral membranes face the visceral endoderm (VE, gray). **b**, Phase, fluorescence, and color-combined images of an E6.5 mouse embryo after microinjection of fluorescein into pre-amniotic cavity. Epiblast is impermeable, but fluorescein diffuses through the gap at the edge of epiblast (border between epiblast and ExE, pink arrow). A and P denote anterior and posterior, respectively. These images are representative of three images from three embryos. **c**, *Top*: Four sagittal sections of an E6.5 embryo stained for DNA, epiblast marker OCT4, and tight junction marker ZO-1. Dotted boxes denote different areas of epiblast, in which ZO-1 intensity was quantified. *Bottom*: Average ZO-1 intensity at the edge (pink) and the rest of epiblast (gray). These images are representative of two sets of images from 2 embryos. Error bars denote SEM. Scale bar 20 μm.

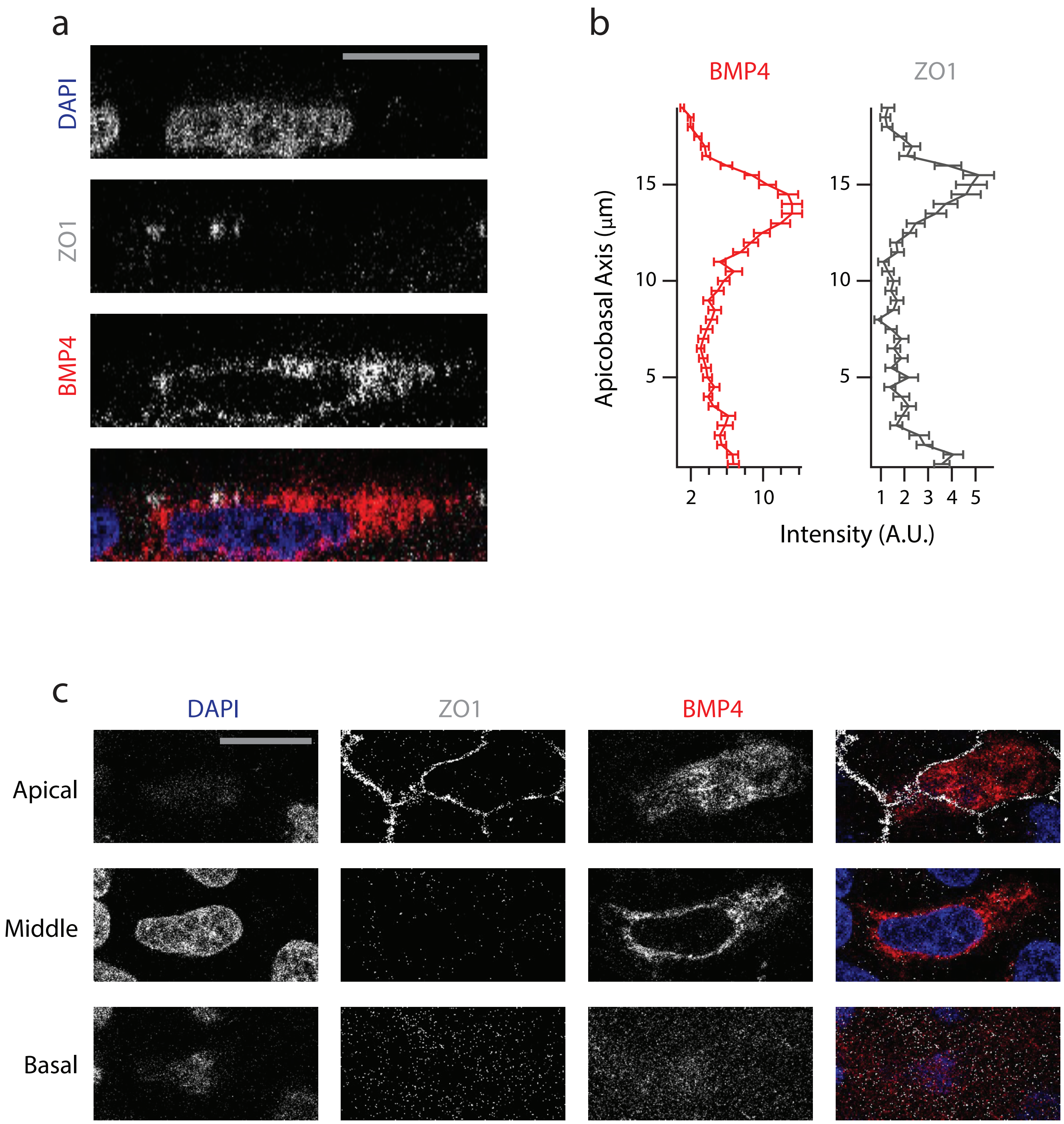

Supplementary Figure 2

Supplementary Fig. 2 | BMP4 localization in hESCs. a, Confocal images in lateral view of a hESC transfected with plasmid expressing GFP-BMP4 (red), immunostained for DNA (blue) and ZO-1 (white). **b,** Plots of GFP-BMP4 (left) and ZO-1 (right) intensity along the apicobasal axis show presence of GFP-BMP4 near apical membrane (n=6 cells from 3 experiments). **c,** Apical, middle, and basal confocal stacks of a transfected hESC immunostained as in **(a).** Error bars denote SEM. Scale bar 10 μm.

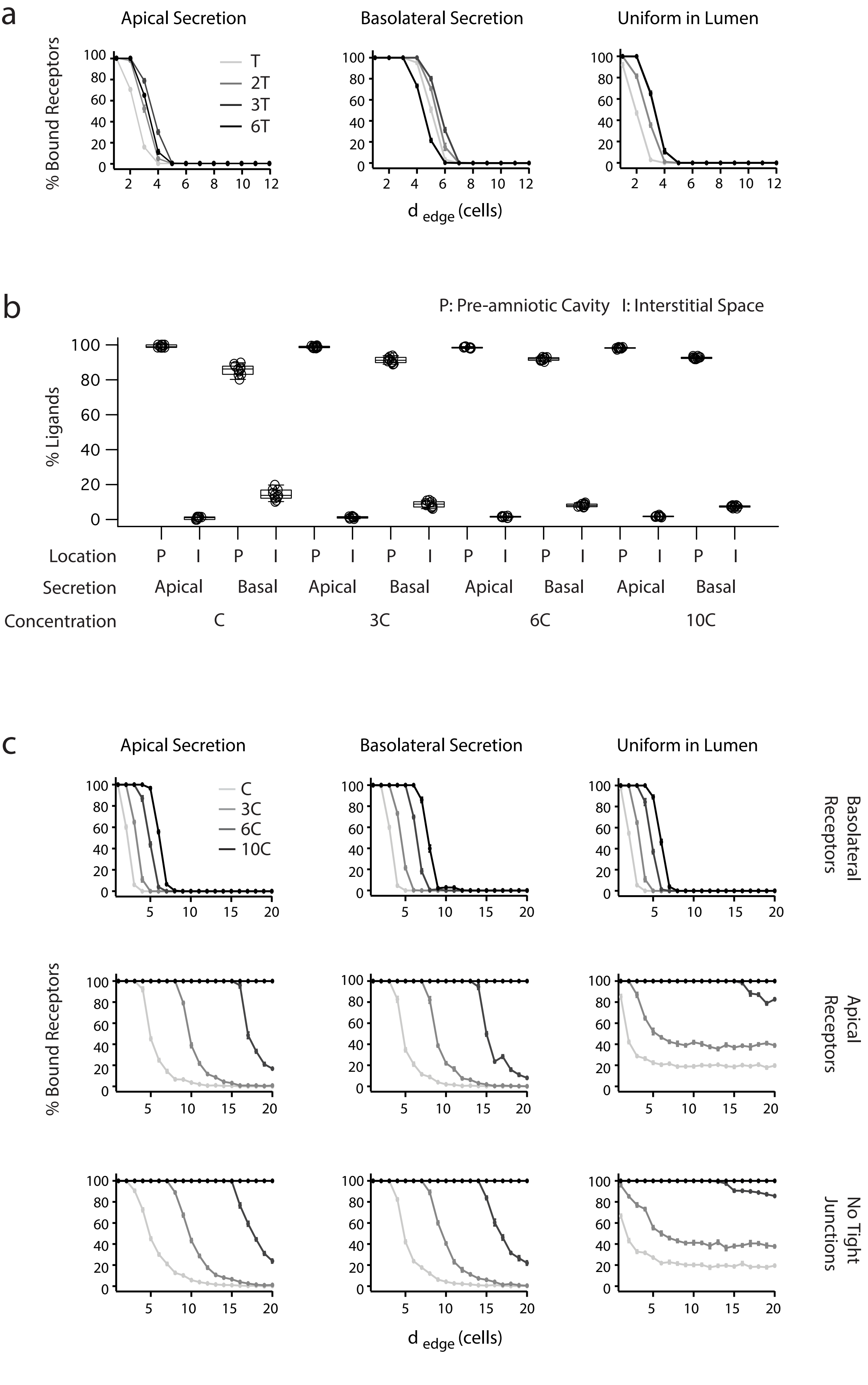

Supplementary Figure 3

Supplementary Fig. 3 | Formation of robust BMP signaling gradient is insensitive to BMP4 secretion mechanism. **a**, Percentage of ligand-bound receptors as function of distance from epiblast edge, d_{edge}, over time in simulations where ligands are secreted apically (left), secreted basolaterally (middle), or presented uniformly in lumen (right). T=5 min. **b**, Percentage of unbound ligands in pre-amniotic cavity (P) and interstitial space (I) at steady state (30 min) in simulations where ligands are secreted apically or basally from ExE. Here, C=0.32 ng/mL. **c**, Percentage of ligand-bound receptors as function of d_{edge} at steady state in simulations where ligands are secreted apically (left), secreted basolaterally (middle), or presented uniformly in lumen (right). Rows correspond to simulations where receptors are localized basolaterally (top) or apically (center), or where tight junctions are absent (bottom). Error bars denote SEM.

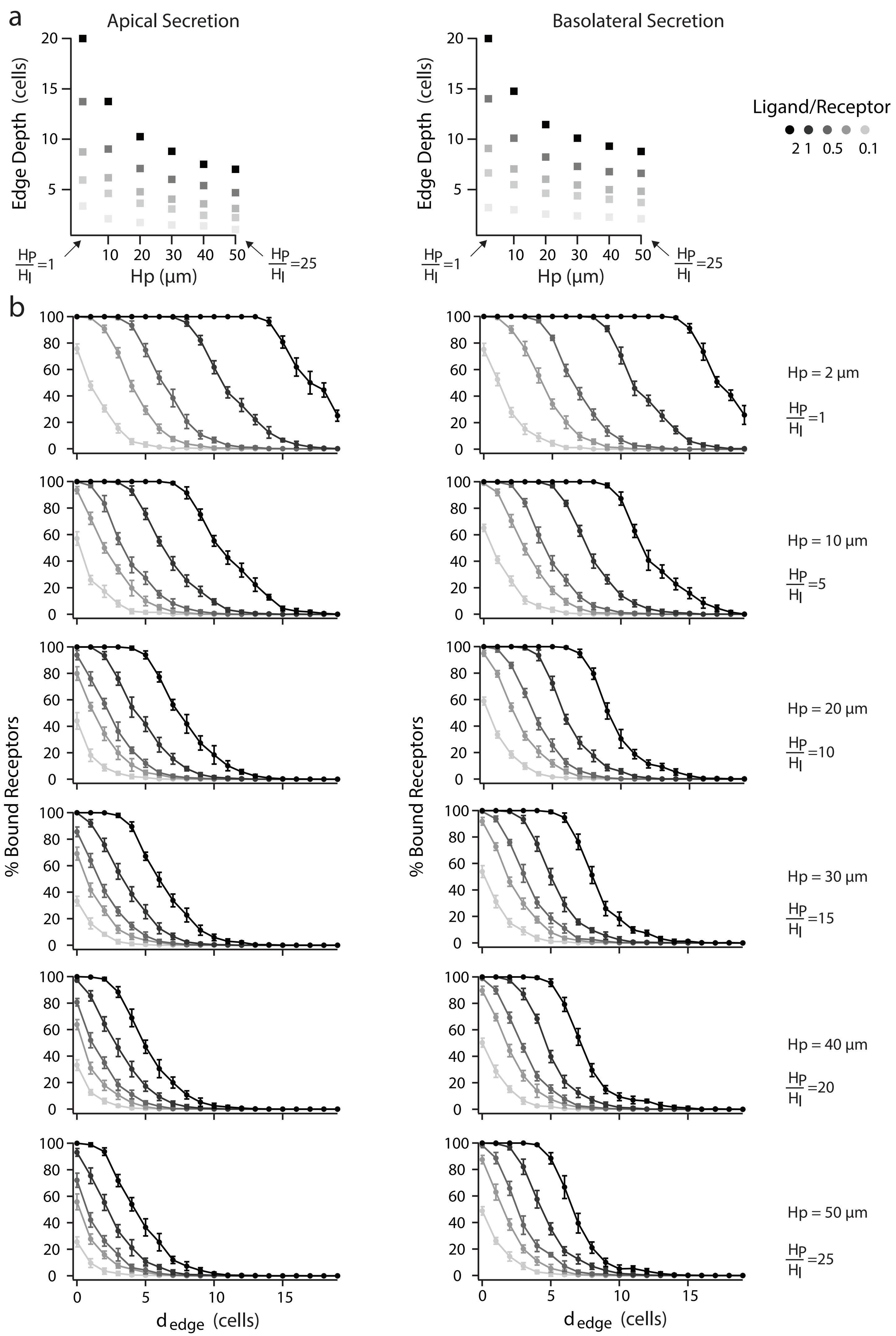

Supplementary Figure 4

Supplementary Fig. 4 | BMP signaling gradient as a function of pre-amniotic cavity size. a, Variation of steady state (90 min) BMP signaling depth as a function of pre-amniotic cavity height (H_p) in simulations where ligands are secreted apically (left) or basolaterally (right) from ExE. Darkness of points and curves corresponds to increased ratio of secreted ligands to receptors. **b**, Percentage of ligand-bound receptors as a function of distance from epiblast edge, d_{edge}, in simulations at steady state where ligands are secreted apically (left) or basolaterally (right). Rows correspond to different pre-amniotic cavity heights. Error bars denote SEM.

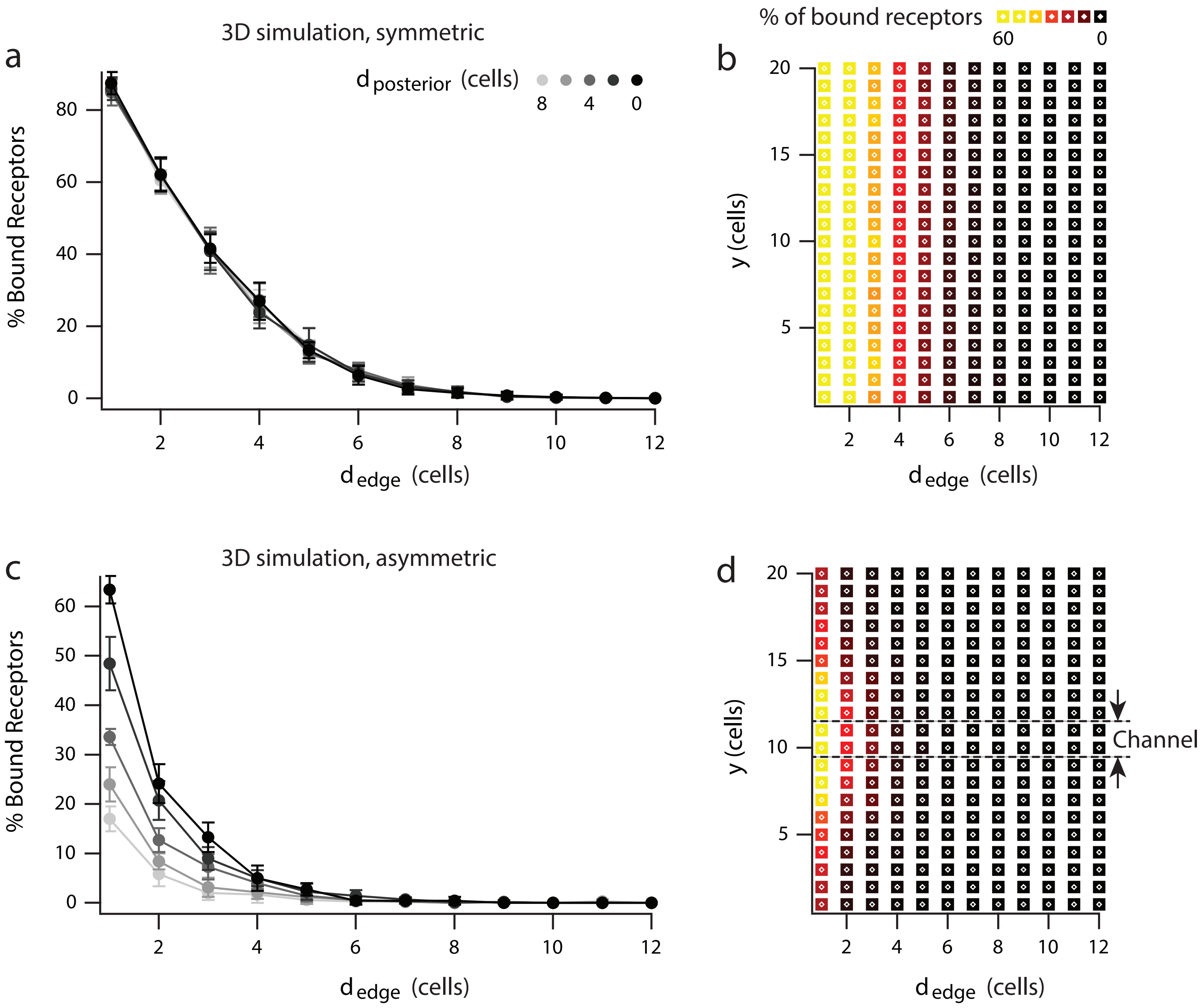

Supplementary Figure 5

Supplementary Fig. 5 | BMP signaling gradient forms from epiblast edge in symmetric and asymmetric 3D simulations a, Percentage of ligand-bound receptors as a function of distance from epiblast edge, d_{edge}, at steady state (90 min) for radially symmetric three-dimensional (3D) simulations, in which channel between epiblast and ExE is present around the embryo. Here ligand/receptor ratio is 0.5. Darkness of curve corresponds to shorter distance of cells from posterior end of embryo ($d_{posterior}$). **b,** Percentage of ligand-bound receptors as a function of d_{edge} and the distance along the anteroposterior axis (y) for individual epiblast cells in symmetric 3D simulations at steady state. **c, d,** Same as in **(a, b)** but for asymmetric 3D simulations in which channel between epiblast and ExE is restricted to posterior end, as seen in fluorescein injection experiments (Supplementary Fig. 1b). Dashed lines mark where channel is open. Error bars denote SEM.

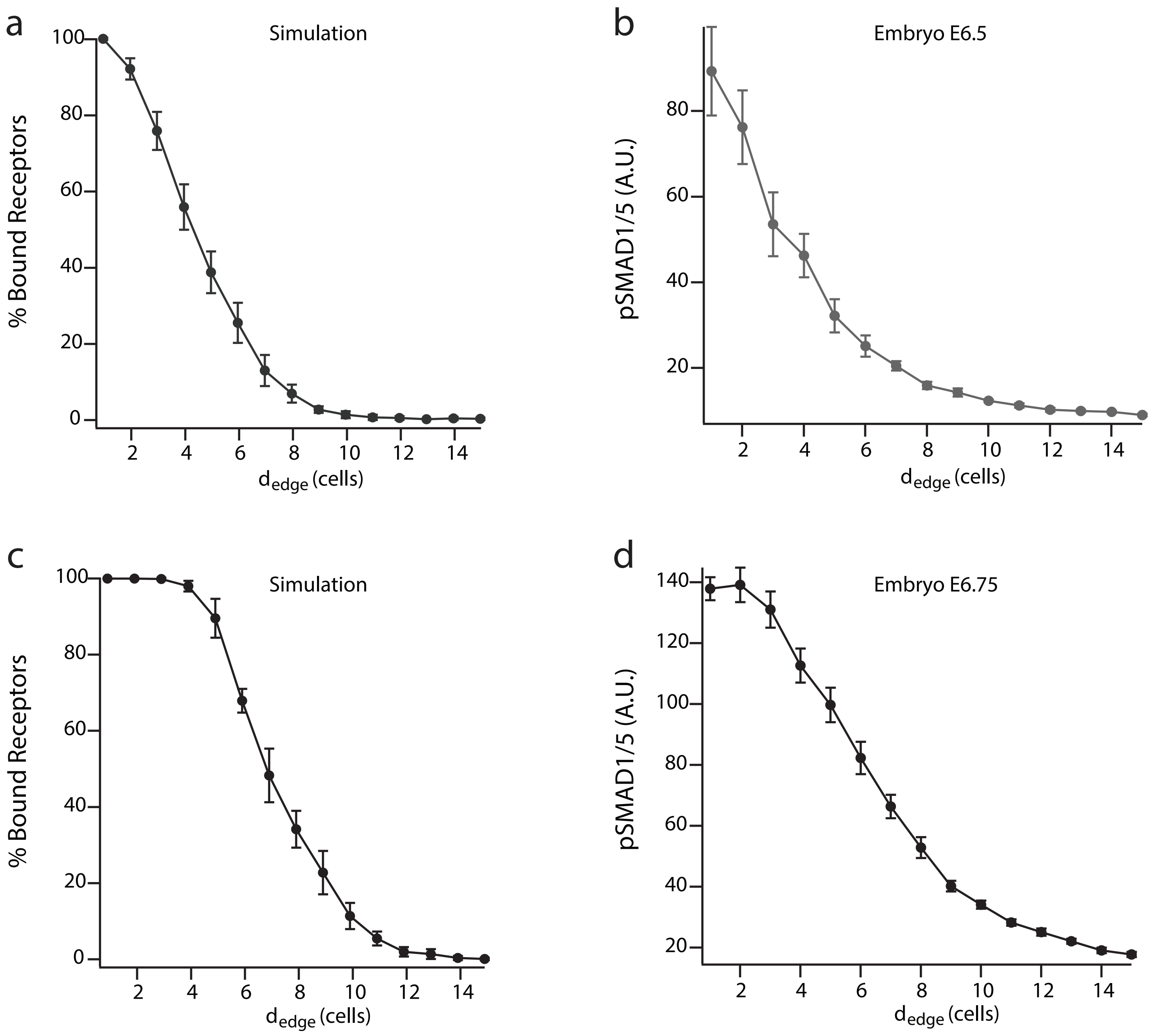

Supplementary Figure 6

Supplementary Fig. 6 | Simulated BMP signaling gradient resembles BMP signaling gradient in mouse embryos a, Percentage of ligand-bound receptors at steady state (90 min) as a function of distance from epiblast edge, d_{edge}, in simulation with apically secreted ligands, as in Fig. 1f. Here ligand-receptor ratio is 1.0. **b,** pSMAD1/5 intensity of epiblast cells as a function of d_{edge} in E6.5 mouse embryo, as in Fig. 3h. Here, d_{edge} represents distance of cell from posterior proximal edge of the epiblast. **c,** Same as **(a)** but for ligand-receptor ratio of 2.0. **d,** Same as **(b)** but for E6.75 mouse embryo, as in Fig. 3f. Error bars denote SEM.

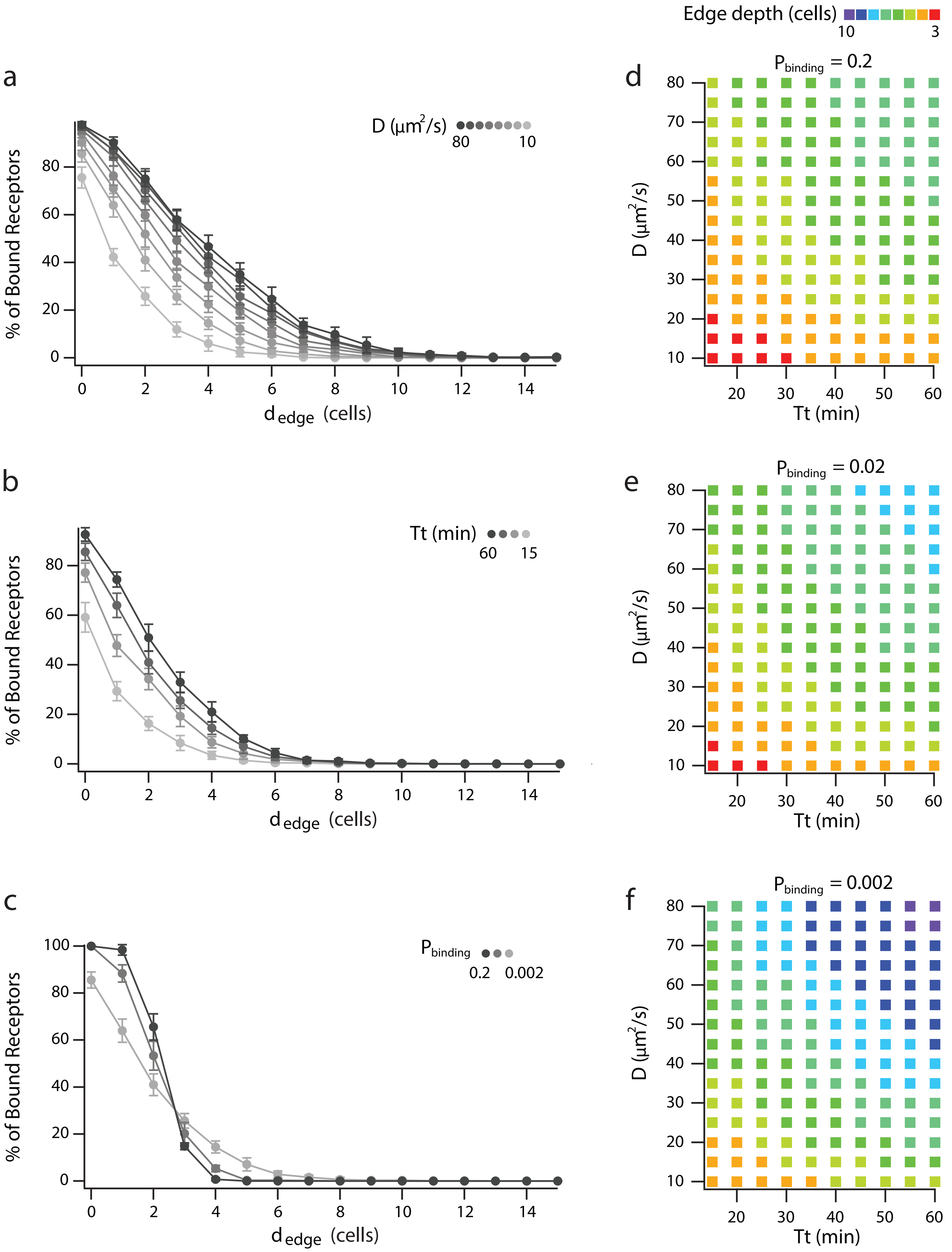

Supplementary Figure 7

Supplementary Fig. 7 | BMP signaling gradient as a function of simulation parameters. a, Percentage of ligand-bound receptors as a function of distance from epiblast edge, d_{edge}, in simulations at steady state (90 min) with different BMP4 diffusion coefficient, D. Here, D is varied from 10 (lightest) to 80 $\mu m^2/s$ (darkest). **b,** Percentage of ligand-bound receptors as a function of d_{edge} in simulations at steady state with different turnover times of ligand-receptor pairs, T_t. Here, T_t is varied from 15 min (lightest) to 60 min (darkest). **c,** Percentage of ligand-bound receptors as a function of d_{edge} in simulations at steady state with different probability of ligand binding to an unbound receptor upon contact ($P_{binding}$). Here, $P_{binding}$ is varied from a diffusion-limited regime (dark) to a regime that is not diffusion-limited (light). **d,** BMP signaling depth as a function of D and T_t in simulations at steady state in which $P_{binding}$=0.2. **e,** Same as **(d)** but for $P_{binding}$=0.02. **f,** Same as **(d)** but for $P_{binding}$ =0.002. Error bars denote SEM. Here ligand-receptor ratio is 0.5.

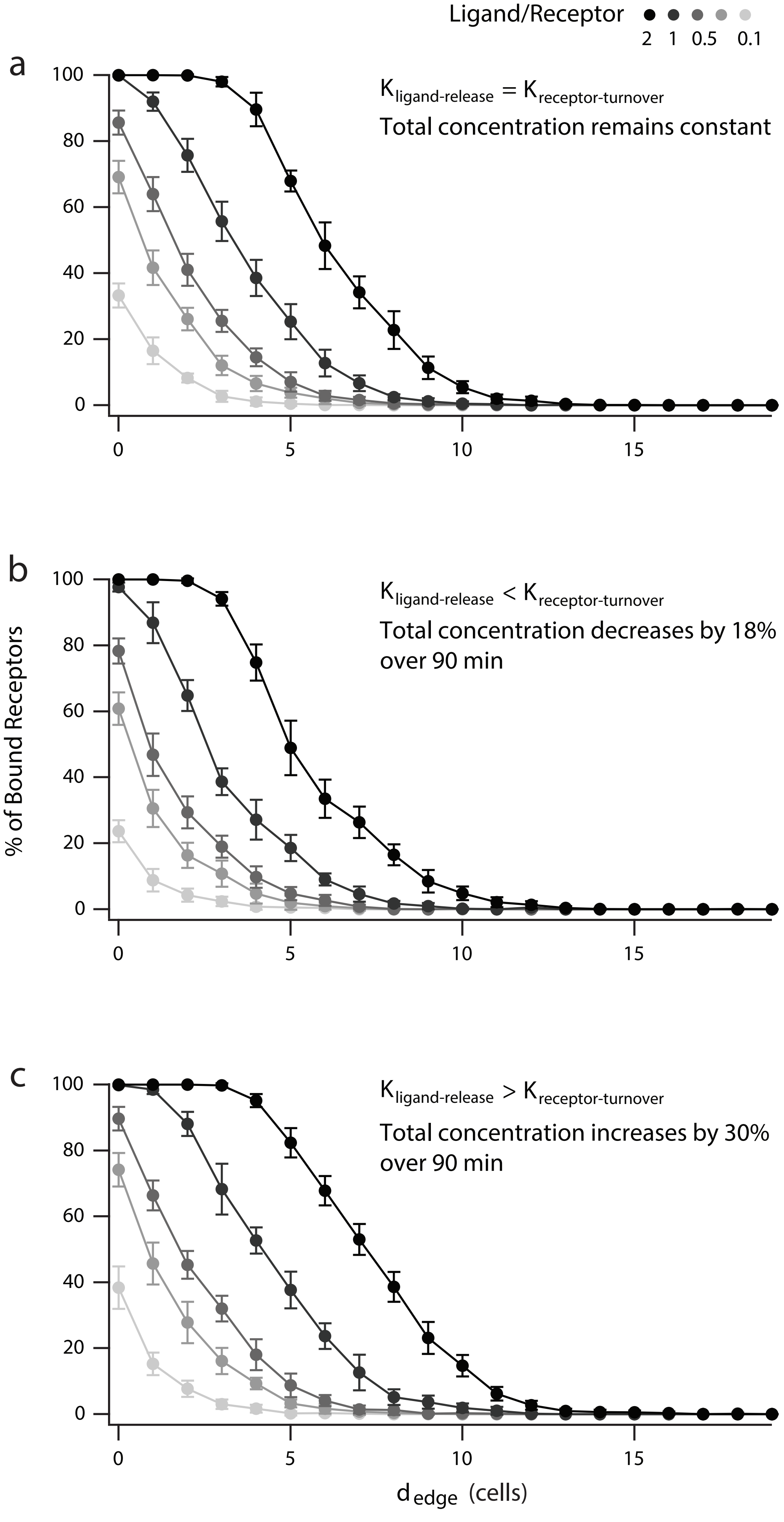

Supplementary Figure 8

Supplementary Fig. 8 | BMP signaling gradient in equilibrium and non-equilibrium simulations. a, Percentage of ligand-bound receptors as a function of distance from epiblast edge, d_{edge}, in simulations at steady state (90 min). Ligand secretion and ligand-receptor turnover rates are coupled so that the total number of ligands remains constant. Darkness of points and curves corresponds to increased ratio of secreted ligands to receptors. **b,** Same as in **(a)** but for non-equilibrium simulation at T=90 min in which total ligand number steadily decreases. Here, no new ligands are secreted after an initial burst at T=0 min. **c,** Same as in **(a)** but for non-equilibrium simulation at T=90 min in which total ligand number steady increases. Here, ligand secretion and ligand-receptor turnover rates are uncoupled, and the secretion rate is significantly higher than the turnover rate. Error bars denote SEM.

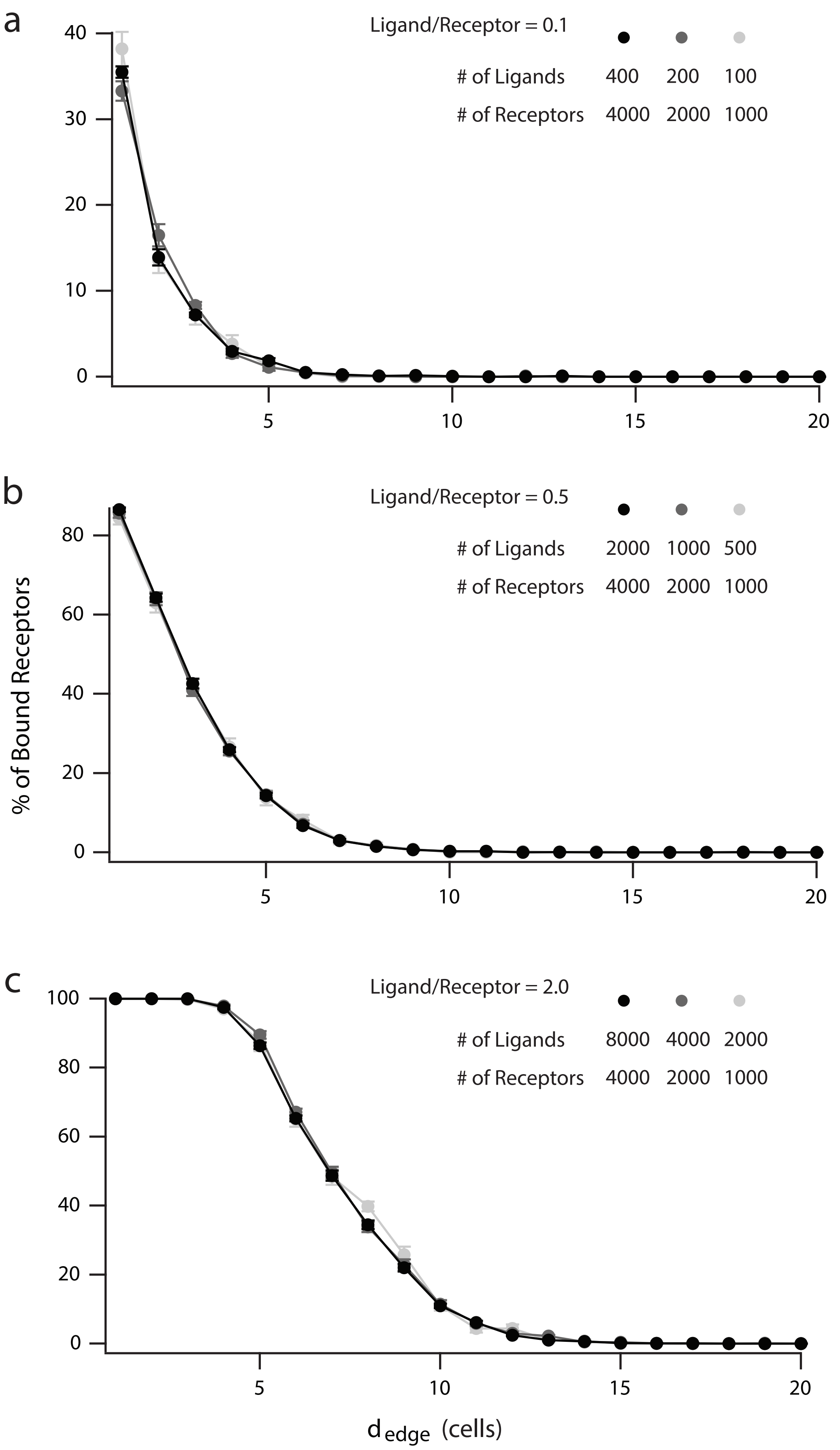

Supplementary Figure 9

Supplementary Fig. 9 | BMP signaling gradient in simulation as a function of total ligand and receptor numbers. a, Percentage of ligand-bound receptors as a function of distance from epiblast edge, d_{edge}, in simulations at steady state (90 min) with a ligand-receptor ratio of 0.1, for different total numbers of ligands and receptors. **b,** Same as **(a)** but for a ligand-receptor ratio of 0.5. **c,** Same as **(a)** but for a ligand-receptor ratio of 2. Error bars denote SEM.

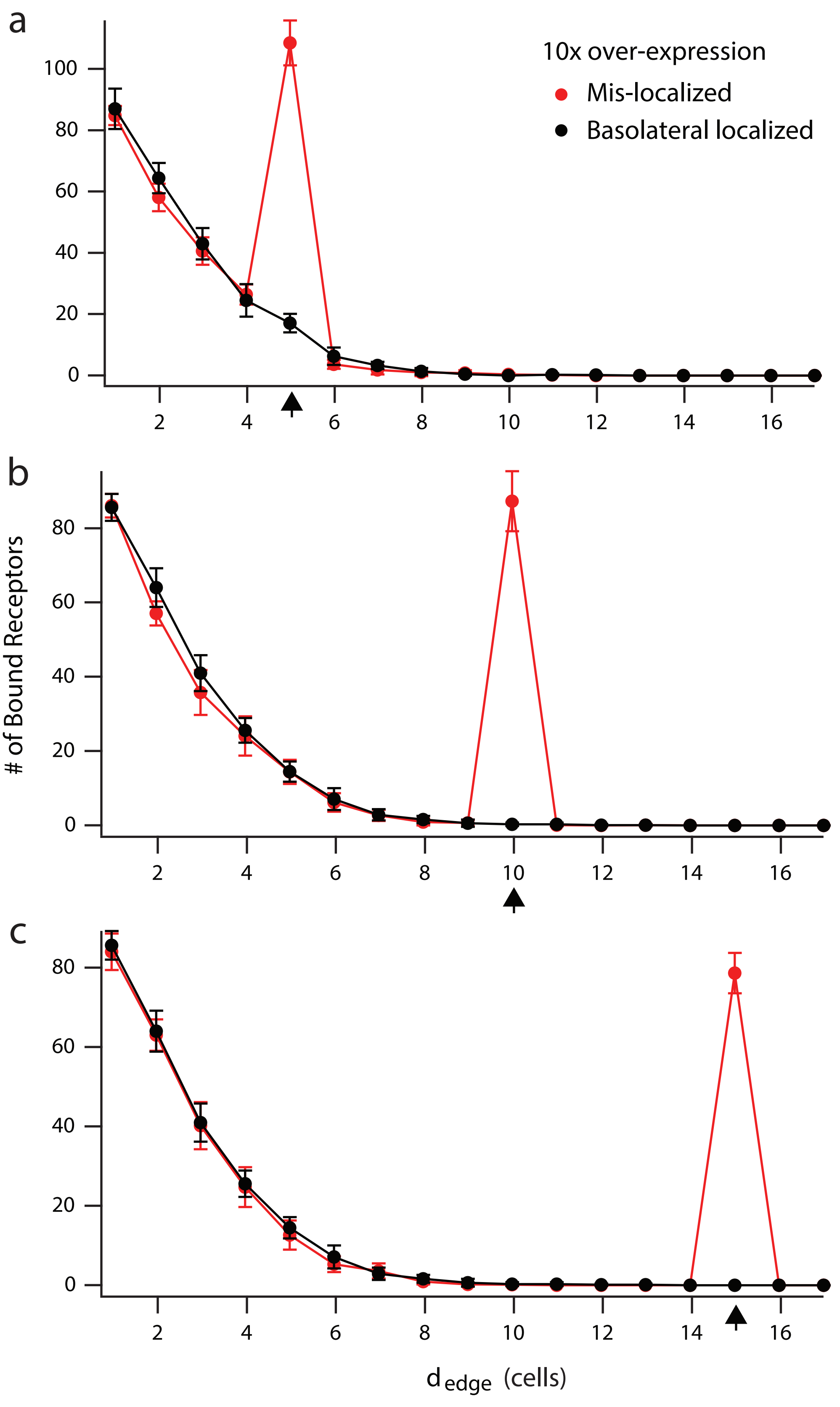

Supplementary Figure 10

Supplementary Fig. 10 | Effect of receptor overexpression and mis-localization on BMP signaling gradient in simulation. a, Number of ligand-bound receptors as a function of distance from epiblast edge, d_{edge}, in simulations at steady state (90 min). In these simulations, a single cell (black arrow) at 5 cell widths from the epiblast edge overexpresses tenfold more wild type receptors that localize basolaterally (black) or mutant receptors that are mis-localize apically and basolaterally (red). For consistency with other figures, number of ligand-bound receptors is rescaled so that "100" corresponds to the default number of receptors in epiblast cells. **b,** Same as **(a)** but receptors are overexpressed in a cell at 10 cell widths from epiblast edge. **c,** Same as **(a)** but receptors are overexpressed in a cell at 15 cell widths from epiblast edge. Error bars denote SEM.

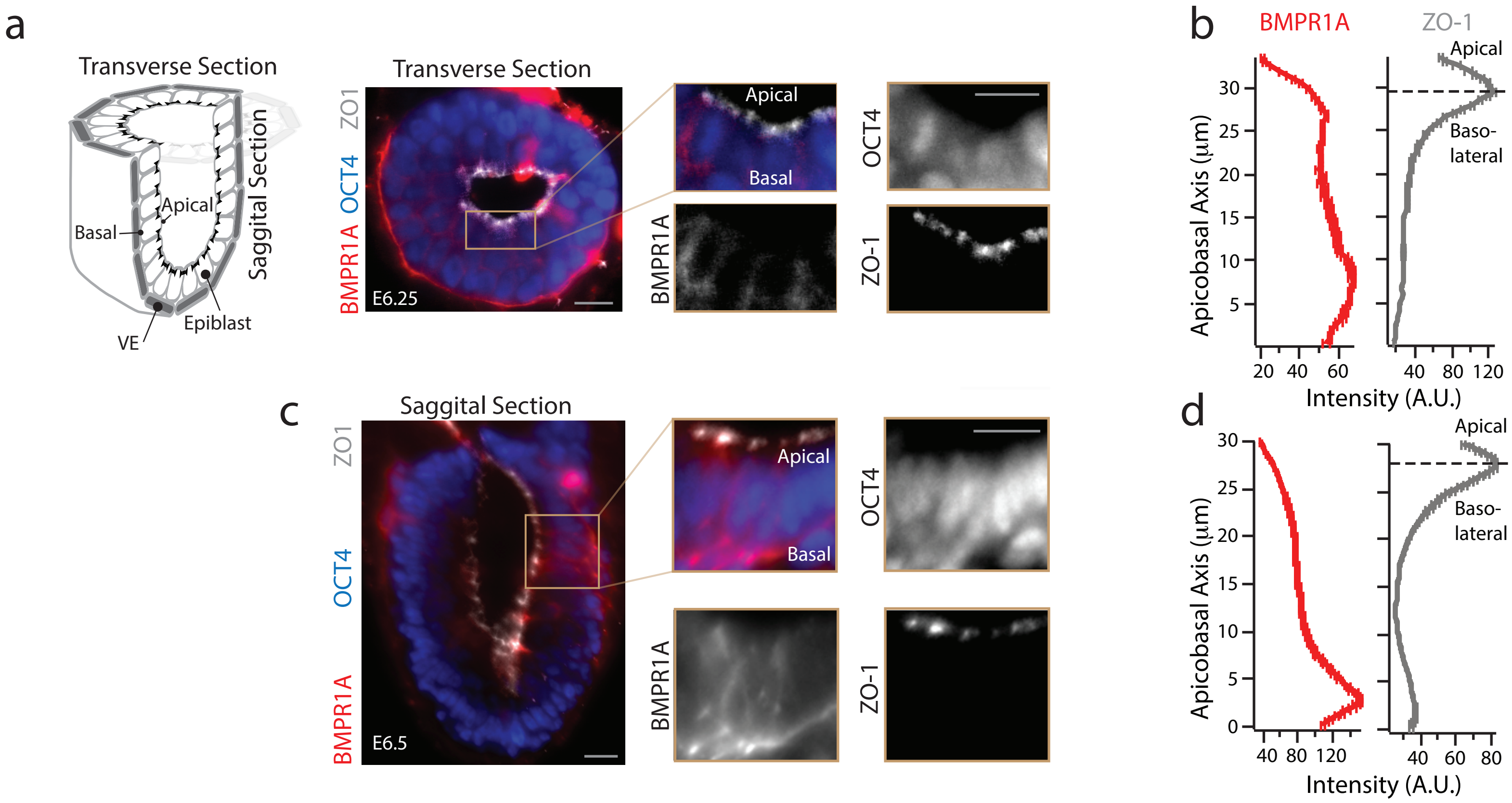

Supplementary Figure 11

Supplementary Fig. 11 | TGF-β and BMP receptors localize at basolateral membrane of mouse epiblast *in vivo*. a, Transverse section of an E6.25 mouse embryo stained for OCT4 (blue), BMPR1A (red), and ZO-1 (white) shows receptors localized at basolateral membrane of epiblast. **b**, BMPR1A (left) and ZO-1 (right) levels along apicobasal axis. **c**, **d**, same as (**a**, **b**) but for a sagittal section of an E6.5 mouse embryo. These images are representative of two sets of images from two embryos. Scale bar 20 μm. Error bars denote SEM.

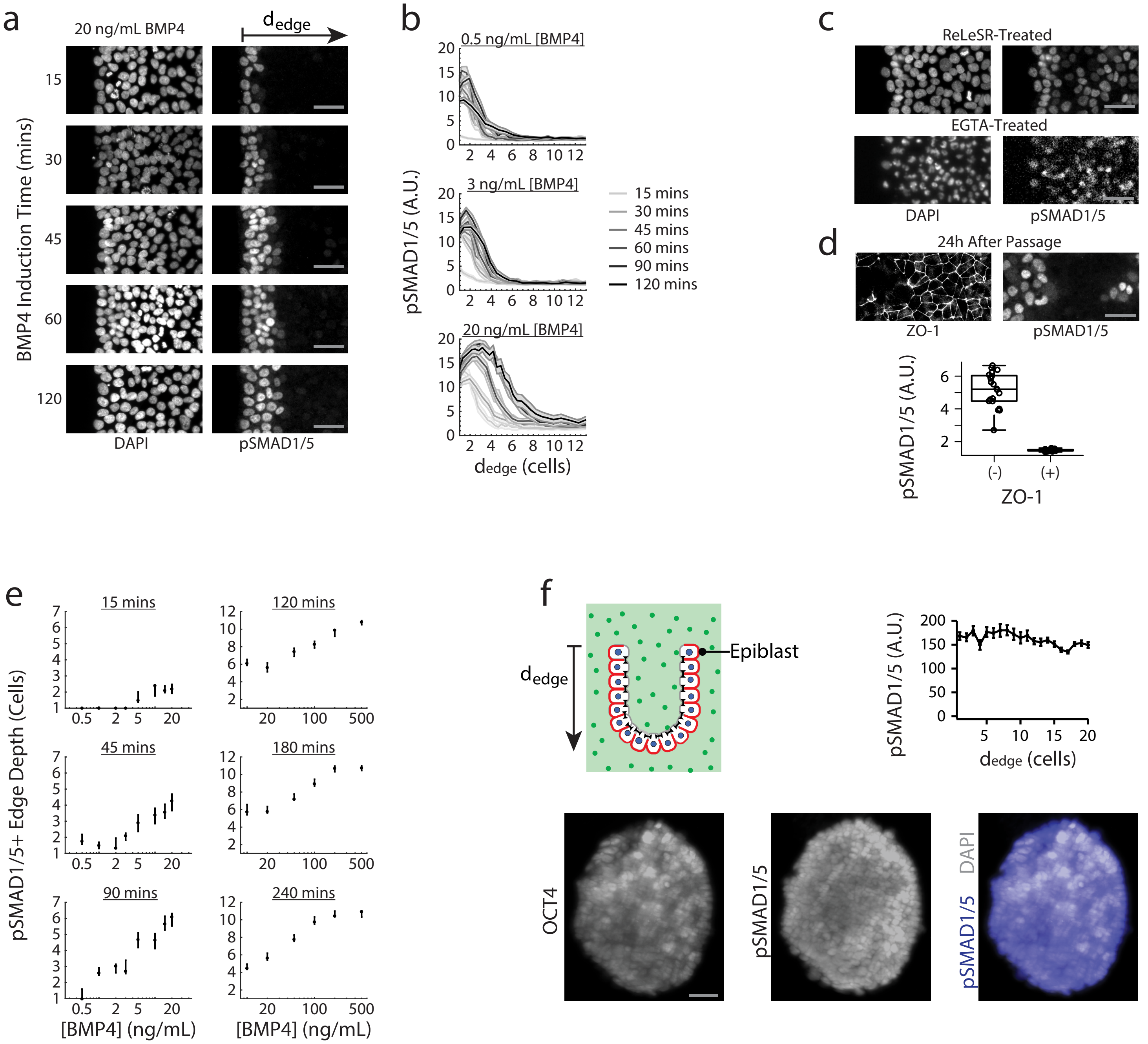

Supplementary Figure 12

Supplementary Fig. 12 | Dependence of BMP signaling gradient on epithelial integrity and embryo geometry. a, hESC colonies treated with 20 ng/mL BMP4 for 15-120 min and stained for DNA and pSMAD1/5. **b**, pSMAD1/5 levels of hESCs as a function of their distance from the nearest colony edge, d_{edge}, after 15-120 min of BMP4 treatment. **c**, hESCs stained for DNA and pSMAD1/5 after treatment with ReLeSR (top) or EGTA (bottom) (see Methods) followed by 20 min BMP4 induction at 5 ng/mL. **d**, *Top*: hESCs exposed to 5 ng/mL BMP4 for 20 min and stained for ZO-1 and pSMAD1/5 at 24 hours after single cell passage. *Bottom*: Average pSMAD1/5 levels of hESCs surrounded by tight junctions (+) and hESCs not surrounded by tight junctions (-), as indicated by ZO-1 immunostain (20 cells of each condition, from 2 experiments). **e**, BMP signaling depth (as indicated by pSMAD1/5 levels of hESCs) as a function of BMP4 concentration after 15-240 min of treatment. In hESC data, error bars denote 95% confidence intervals and scale bar 50 μm. **f**, *Top*: Illustration of mouse epiblast after removal of ExE and VE (see Methods), soaking in media containing 10 ng/mL BMP4. *Bottom*: E6.25 mouse embryo stained for OCT4 and pSMAD1/5 shows BMP4 concentration is sufficient to induce signaling activity in all epiblast cells if ExE and VE are removed. *Top right*: Average pSMAD1/5 level of epiblast cells as function of d_{edge} in soaked embryos with ExE and VE removed. Here, d_{edge} represents cell's distance from proximal edge of epiblast (10 cells each condition, from 1 experiment). In mouse data, error bars denote SEM and scale bar 20 μm.

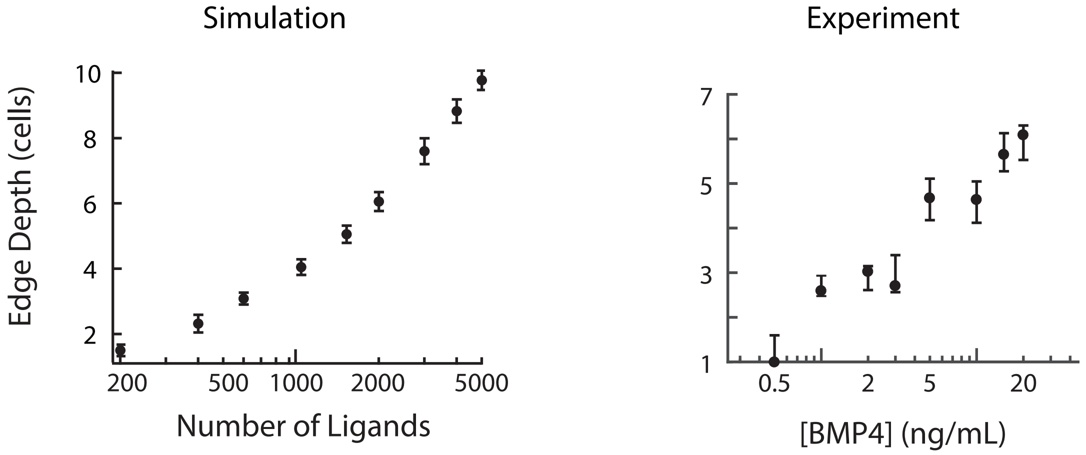

Supplementary Figure 13

Supplementary Fig. 13 | BMP signaling depth in simulation and in hESCs. BMP signaling depth from epithelial edge as a function of BMP4 concentration in mouse embryo simulation (left) and in hESC colonies (right) after 90 min of exposure. The BMP signaling depth is defined by 50% of maximum BMP4 signaling. Simulation error bars denote SEM, hESC data error bars denote 95% confidence intervals.

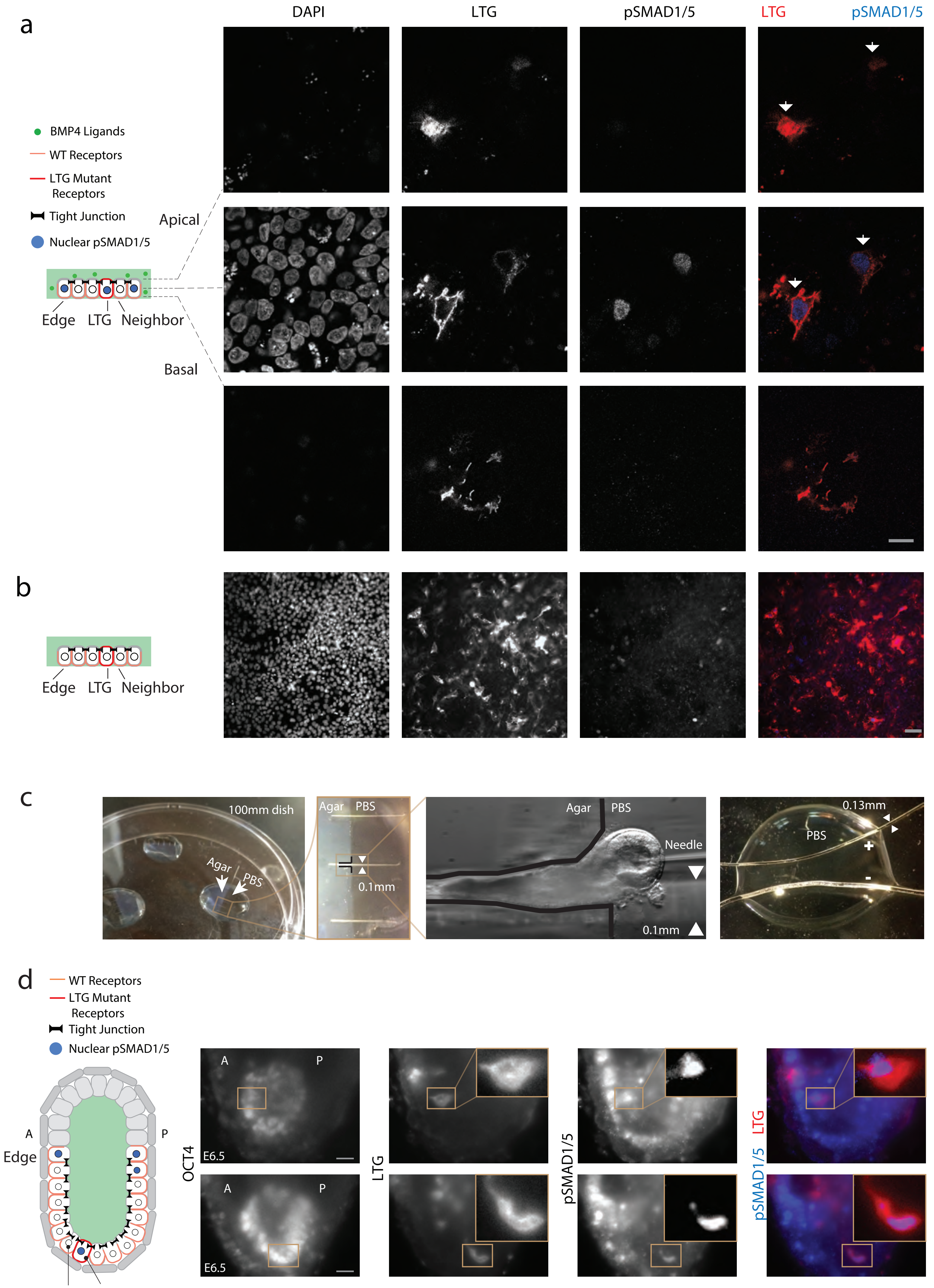

Supplementary Figure 14

Supplementary Fig. 14 | Receptor mis-localization results in ectopic BMP signaling *in vitro* and *in vivo*. **a**, Apical (top row), middle (middle row), and basal (bottom row) confocal image stacks of hESC colony transfected with mutant receptor plasmid (BMPR1A^{A514G}-Clover-IRES-BMPR2^{A494G}) and exposed to 10 ng/mL BMP4 for 30 min. Two cells in center of colony expressed mis-localized BMP receptors. (*Left to Right*): DNA, Clover (LTG mutant receptors; red), pSMAD1/5 (blue), and color-combined channels. These images show that receptor mis-localization leads to ectopic pSMAD1/5 activation in the center of hESC colonies. Scale bar 10 μm. These images are representative of 8 images from 2 experiments. **b**, Same as **(a)** but without BMP4 induction. These images show that the expression of mutant receptors in the absence of exogenous BMP4 ligands does not result in ectopic pSMAD1/5 activation. Scale bar 40 μm. These images are representative of 4 images from 2 experiments. **c**, Custom-made device for microinjection (left) and electroporation (right) of mouse embryos (see Methods). **d**, *Left*: Illustration of mouse embryo with a single cell expressing mis-localized LTG mutant BMP receptors, leading to ectopic pSMAD1/5 activity. A and P denote anterior and posterior, respectively. *Right*: Two E6.5 mouse embryos transfected with mutant receptor plasmid and immunostained for OCT4, Clover (LTG mutant receptors, red), and pSMAD1/5 (blue). Insets show transfected cells at increased magnification and contrast. Cells expressing mutant receptors have ectopic pSMAD1/5 activity in both the anterior (top row) and distal (bottom row) epiblast. These images are representative of 13 images from 13 experiments. Scale bar 20 μm.

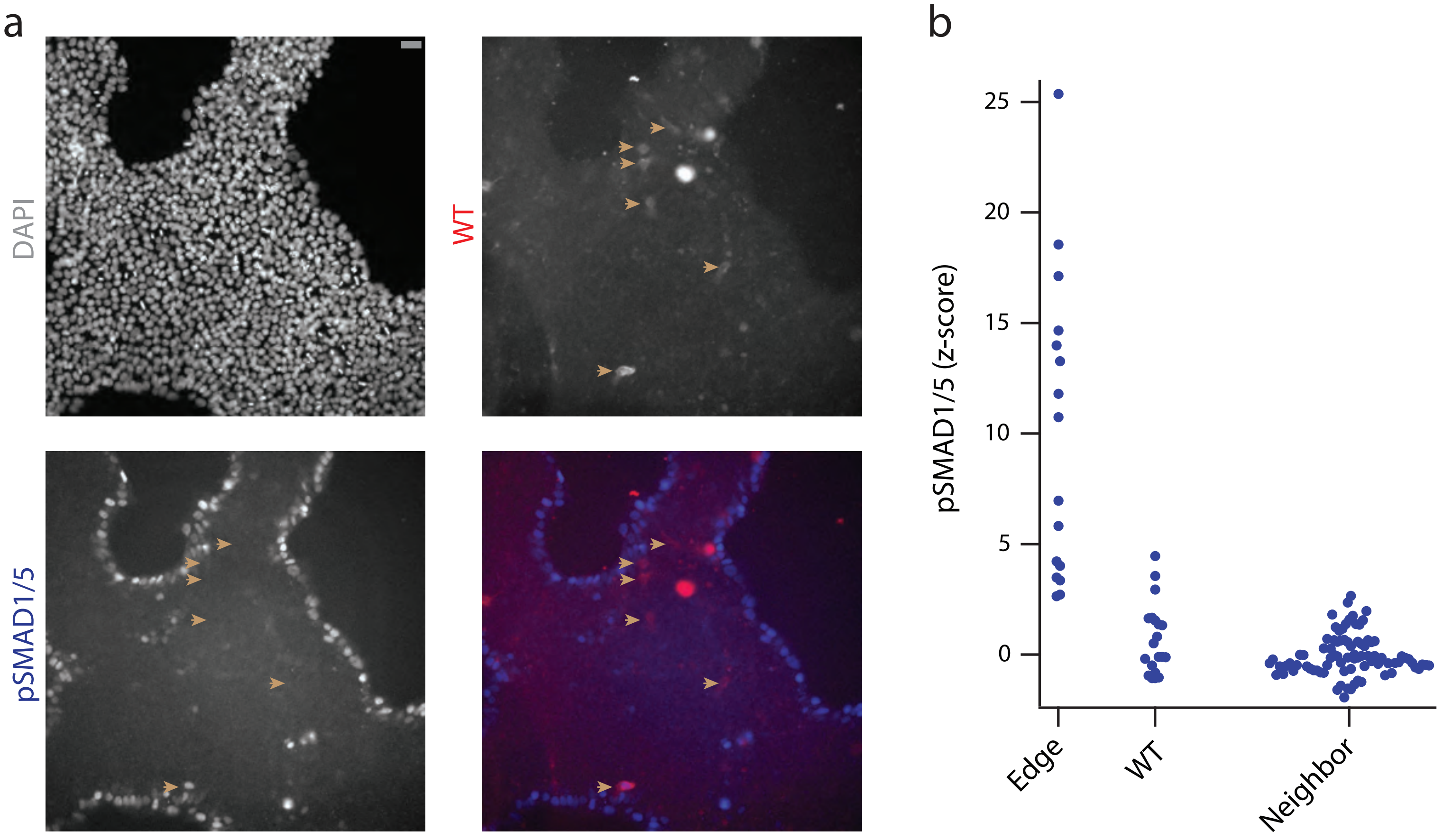

Supplementary Figure 15

Supplementary Fig. 15 | Effect of wild type receptor overexpression on BMP signaling. a, hESC colony transfected with wild type receptor plasmid (BMPR1A-Clover-IRES-BMPR2), exposed to 10 ng/ml BMP4 for 30 min, and stained for DNA and pSMAD1/5 (blue). Brown arrows indicate transfected cells expressing wild type receptors (WT, red). **b,** pSMAD1/5 intensities of hESCs after 30 min BMP4 induction: cells at colony edge (Edge, n=21 in 2 experiments); non-edge cells overexpressing wild type BMPR1A and BMPR2 receptors (WT, n=21 in 2 experiments); and non-transfected neighbors of transfected cells (Neighbor, n=86 in 2 experiments). Z-score denotes number of standard deviations beyond background mean (of neighboring non-transfected cells). Edge (n=6) and WT cells (n=1) with z-score greater than 25 are not shown. Scale bar 20 μm.

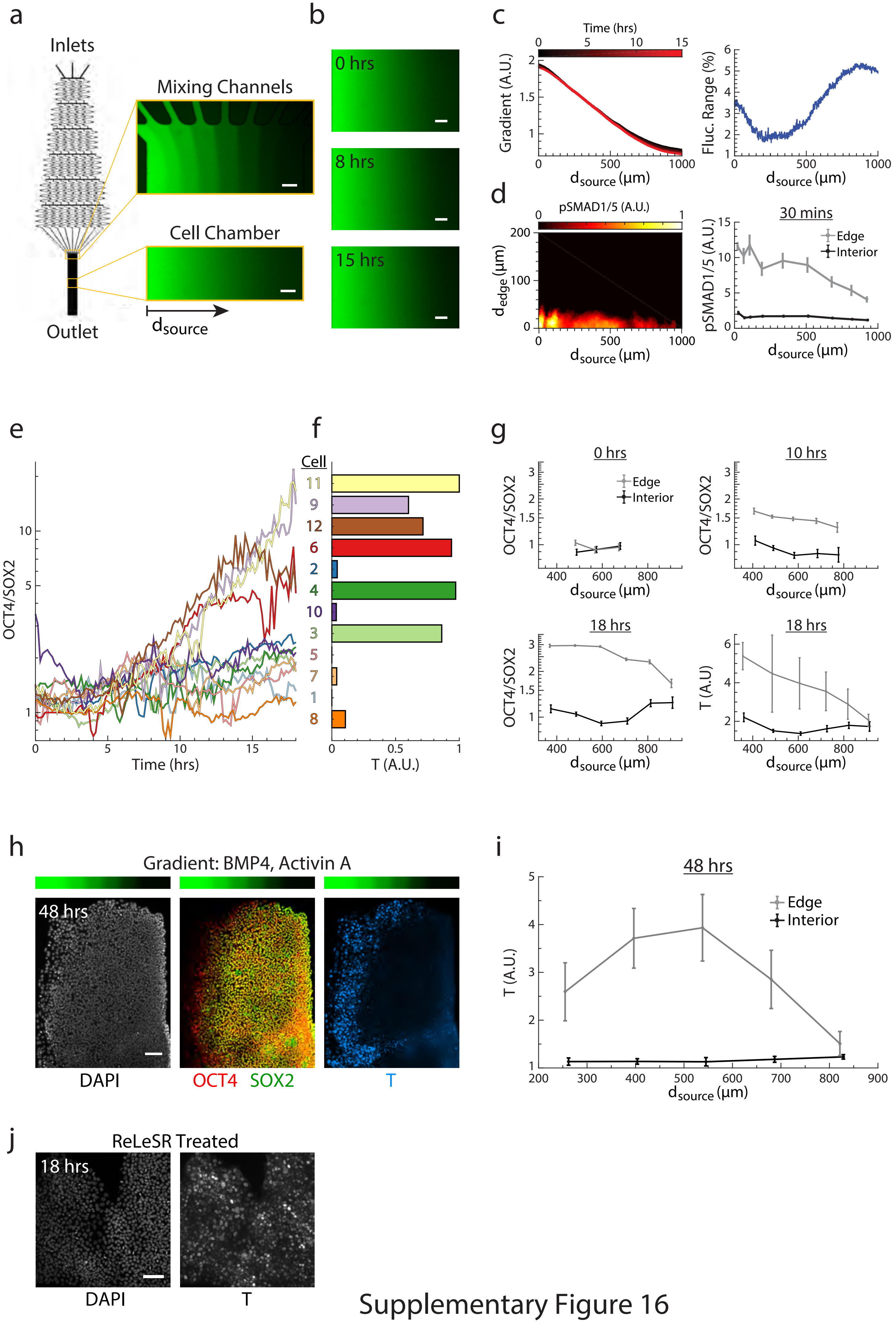

Supplementary Figure 16

Supplementary Fig. 16 | Distance from tissue edge and distance from signal source govern signaling and patterning of epithelial tissue. **a**, Diagram of microfluidic device. (Inset) Visualization of fluorescein gradient at top and middle of cell chamber in microfluidic device. **b**, Visualization of microfluidic gradient after 0, 8, and 15 hours of flow. **c**, Level of fluorescein gradient over 15 hours (left) and its maximum range of fluctuation (right) across cell chamber as function of distance from left side of the chamber (d_{source}). Fluctuation range is given as percentage of total gradient range. **d**, (Left) pSMAD1/5 levels of hESCs exposed to BMP4 gradient for 30 min as a function of d_{source} and d_{edge}. (Right) pSMAD1/5 levels of edge and interior hESCs as a function of d_{source}. Edge cells are defined as cells within 2 cell widths of a colony edge, while interior cells are those further than 6 cell widths from the nearest colony edge. **e**, OCT4/SOX2 ratios of 12 tracked cells from Fig. 5e over time-lapse experiment. **f**, BRACHYURY/T levels of 12 tracked cells from Fig. 5e at end of time-lapse experiment. **g**, OCT4/SOX2 ratios at 0, 10, and 18 hours and BRACHYURY/T levels at 18 hours during time-lapse differentiation of edge and interior hESCs as a function of d_{source} (n=1,275 cells). **h**, OCT4-RFP SOX2-YFP hESCs after 48 hours of differentiation in BMP4 and ACTIVIN A gradient (above) as in Fig. 5e, stained for DNA and BRACHYURY/T. **i**, BRACHYURY/T levels of edge and interior hESCs after 48-hour gradient differentiation as a function of d_{source}. Here, edge cells are defined as cells within 126 μm of colony edge, while interior cells are those further than 283 μm from nearest colony edge. **j**, ReLeSR-treated hESCs exposed to uniform BMP4 and ACTIVIN A (10 ng/mL of each) for 18 hours and stained for DNA and BRACHYURY/T. hESCs were treated with ReLeSR for 1 min after 0, 4, and 8 hours of differentiation. The images are representative of 4 images from 2 experiments. Error bars denote 95% confidence intervals and scale bar 100 μm.

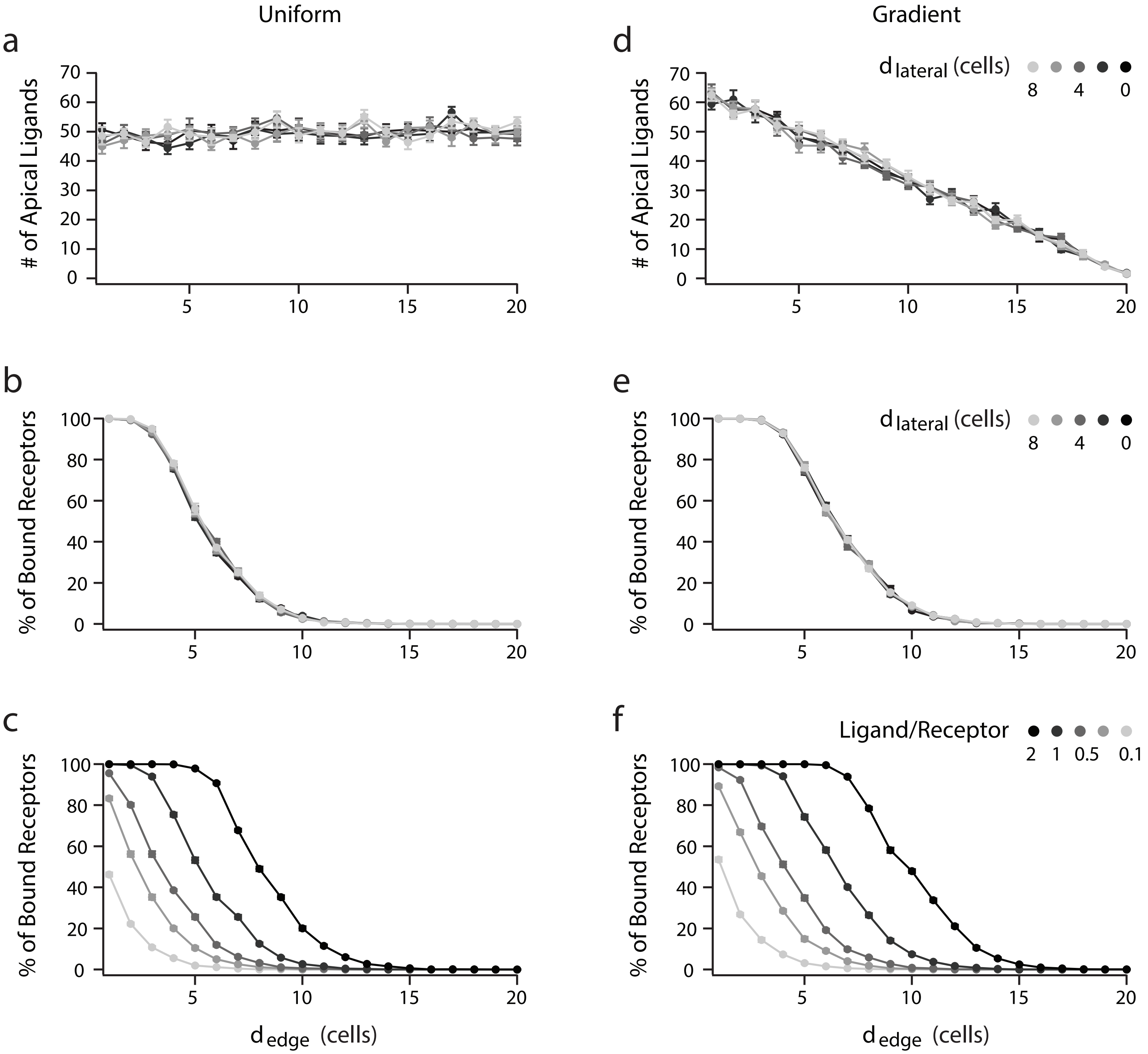

Supplementary Figure 17

Supplementary Fig. 17 | BMP signaling gradient in simulations based on hESC colony geometry *in vitro*. a, Number of apical ligands as a function of distance from nearest colony edge, d_{edge}, in simulations of epithelial hESC colonies exposed to uniform concentration of BMP4 ligands in culture media. Colors of points and curves correspond to position along lateral axis of colony ($d_{lateral}$) perpendicular to d_{edge} axis. **b,** Percentage of ligand-bound receptors as a function of d_{edge}, in simulations from **(a)**. **c,** Percentage of ligand-bound receptors as a function of d_{edge} in simulations from (**a**), where darkness of points and curves corresponds to higher ligand-receptor ratio. Here ligand-receptor ratio of 0.1 correspond to approximately BMP4 concentration of 0.16 ng/ml. **d-f,** Same as **(a-c)** but for simulations of hESC colonies exposed to gradient of BMP4 ligands along d_{edge} axis. Error bars denote SEM.

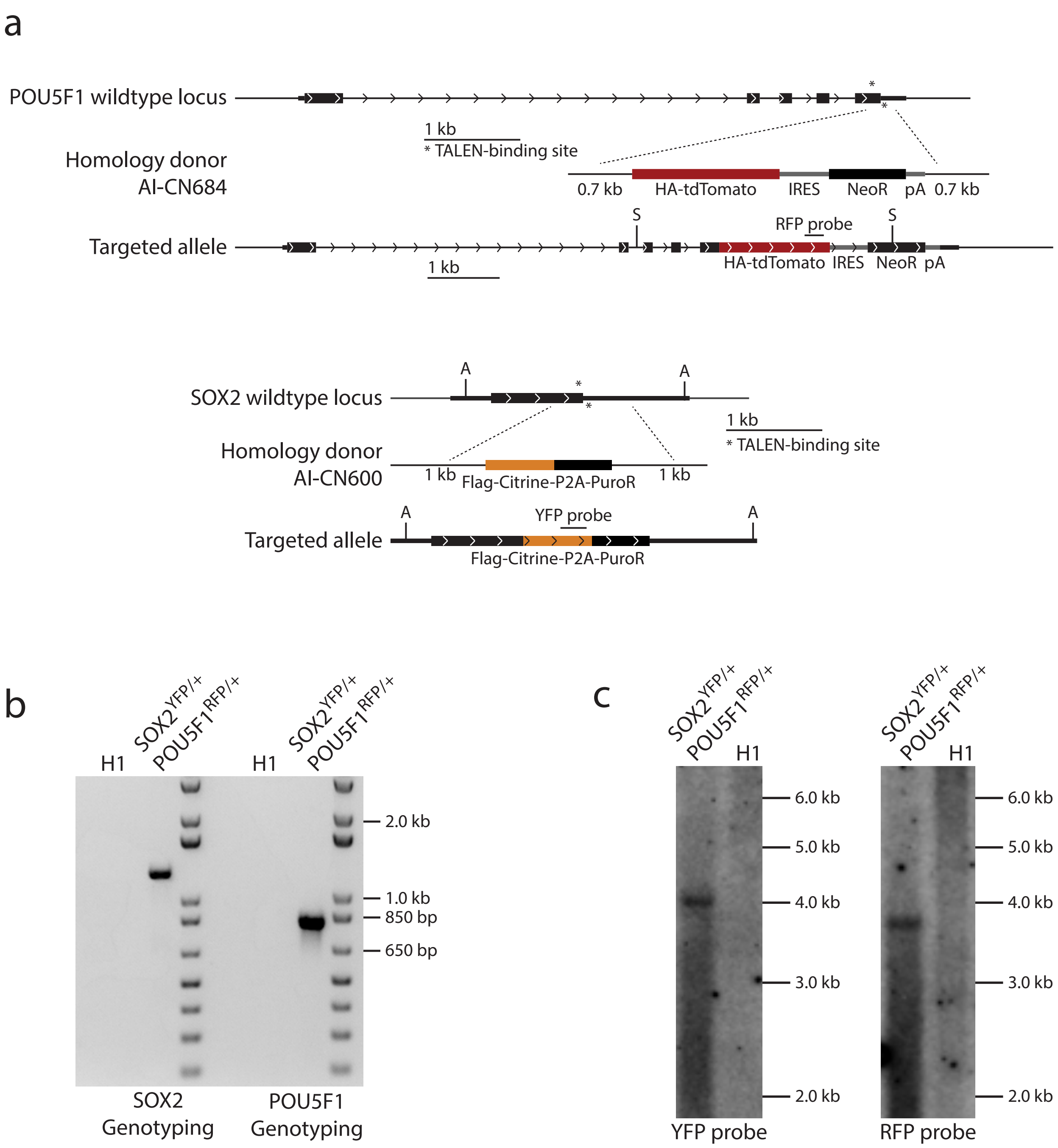

Supplementary Figure 18

Supplementary Fig. 18 | Transgenic *SOX2*$^{YFP/+}$*POU5F1*$^{RFP/+}$ H1 human embryonic stem cell reporter line. **a**, Targeting strategy to generate *SOX2*$^{YFP/+}$*POU5F1*$^{RFP/+}$ reporter cell line. **b**, 5' junction genotyping PCR of the resulting line. **c**, Southern blots of the resulting line. Restriction enzyme sites (A, AflII; S, SacI) are indicated. POU5F1 is also known as OCT4.

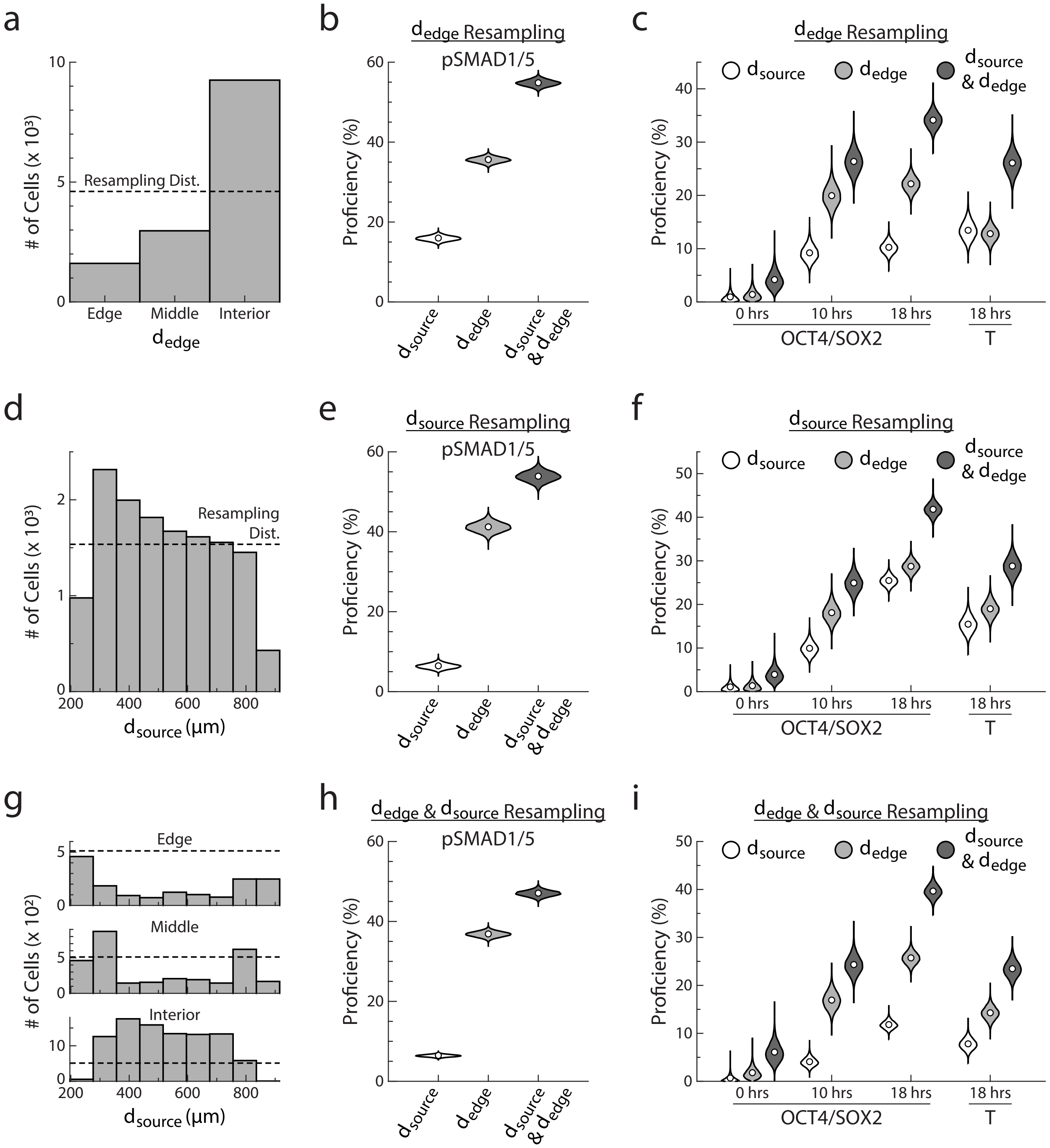

Supplementary Figure 19

Supplementary Fig. 19 | Proficiencies do not change significantly after resampling. a, Distribution of edge cells (d_{edge} < 2 cell widths), middle cells (2 cell widths < d_{edge} < 6 cell widths), and interior cells (d_{edge} > 6 cell widths) in BMP4 gradient hESC microfluidic experiment (Fig. 5c-d). Dashed line indicates distribution after resampling a uniform number of edge, middle, and interior cells (n = 13,828 cells). **b,** Distribution of cells along d_{source} in BMP4 gradient hESC microfluidic experiment. Dashed line indicates distribution after resampling cells uniformly along d_{source}. **c,** From top to bottom, distribution of edge, middle, and interior cells along d_{source} in BMP4 gradient hESC microfluidic experiment. Dashed lines indicate distribution after resampling cells uniformly along both d_{edge} and d_{source}. **d**, As in Fig. 5d, proficiency of d_{source}, d_{edge}, or both coordinates to predict pSMAD1/5 levels, calculated after resampling uniformly from edge, middle, and interior cells. **e,** Same as in **(d)** but calculated after resampling cells uniformly along d_{source}. **f,** Same as in **(d)** but calculated after resampling cells uniformly along both d_{edge} and d_{source}. **g, h, i,** Same as in **(d-f)** but for OCT4/SOX2 and T proficiencies in microfluidic time-lapse experiment from Fig. 5e-h (n = 1,275 cells). Violin plots denote Gaussian kernel density estimates.
