# Supplementary material for: Mouse embryo geometry drives formation of robust signaling gradients through receptor localization
Source: Nat Commun. 2019 Oct 4;10:4516. doi: 10.1038/s41467-019-12533-7 (PMC6778081; doi:10.1038/s41467-019-12533-7)
Supplement: Supplementary file 3 — Reporting Summary [file 41467_2019_12533_MOESM3_ESM.pdf]

nature research

Corresponding author(s): Sharad Ramanathan

Last updated by author(s): Sep 12, 2019

Reporting Summary

Nature Research wishes to improve the reproducibility of the work that we publish. This form provides structure for consistency and transparency in reporting. For further information on Nature Research policies, see Authors & Referees and the Editorial Policy Checklist.

Statistics

For all statistical analyses, confirm that the following items are present in the figure legend, table legend, main text, or Methods section.

n/a	Confirmed	
[ ]	[x]	The exact sample size (n) for each experimental group/condition, given as a discrete number and unit of measurement
[ ]	[x]	A statement on whether measurements were taken from distinct samples or whether the same sample was measured repeatedly
[ ]	[x]	The statistical test(s) used AND whether they are one- or two-sided<br>*Only common tests should be described solely by name; describe more complex techniques in the Methods section.*
[x]	[ ]	A description of all covariates tested
[x]	[ ]	A description of any assumptions or corrections, such as tests of normality and adjustment for multiple comparisons
[x]	[ ]	A full description of the statistical parameters including central tendency (e.g. means) or other basic estimates (e.g. regression coefficient) AND variation (e.g. standard deviation) or associated estimates of uncertainty (e.g. confidence intervals)
[x]	[ ]	For null hypothesis testing, the test statistic (e.g. F, t, r) with confidence intervals, effect sizes, degrees of freedom and P value noted<br>*Give P values as exact values whenever suitable.*
[x]	[ ]	For Bayesian analysis, information on the choice of priors and Markov chain Monte Carlo settings
[x]	[ ]	For hierarchical and complex designs, identification of the appropriate level for tests and full reporting of outcomes
[x]	[ ]	Estimates of effect sizes (e.g. Cohen's d, Pearson's r), indicating how they were calculated

Our web collection on statistics for biologists contains articles on many of the points above.

Software and code

Policy information about availability of computer code

Data collection	We wrote a C code to simulate BMP4 dynamics in mouse embryo. The link to the code can be found at the code availability section of the Methods.
Data analysis	We wrote Matlab codes to analyze hESCs images. The link to the codes can be found at the code availability section of the Methods.

For manuscripts utilizing custom algorithms or software that are central to the research but not yet described in published literature, software must be made available to editors/reviewers. We strongly encourage code deposition in a community repository (e.g. GitHub). See the Nature Research guidelines for submitting code & software for further information.

Data

Policy information about availability of data

All manuscripts must include a data availability statement. This statement should provide the following information, where applicable:
- Accession codes, unique identifiers, or web links for publicly available datasets
- A list of figures that have associated raw data
- A description of any restrictions on data availability

The authors declare that all data supporting the findings of this study are available within the article and its supplementary information files or from the corresponding author upon reasonable request.

Field-specific reporting

Please select the one below that is the best fit for your research. If you are not sure, read the appropriate sections before making your selection.

[x] Life sciences [ ] Behavioural & social sciences [ ] Ecological, evolutionary & environmental sciences

For a reference copy of the document with all sections, see nature.com/documents/nr-reporting-summary-flat.pdf

Life sciences study design

All studies must disclose on these points even when the disclosure is negative.

Sample size	Sample size was determined by our previous experience on stem cells, and work of other groups on receptor localization, stem cells, and moue embryos.
Data exclusions	As we stated in the "Plasmid construction and transient expression of receptors" section of the Methods, we excluded cells with excessive levels of expression, aggregations of fluorescent proteins, and membrane blebbing. The rational is to minimize side effects caused by plasmid expression of tagged protein.<br>As stated in the "Mouse embryo culture" section of the Methods, we excluded embryos with visible defects. The rationale is to minimize suboptimal culture conditions as well as damages to embryos during dissection, microinjection, and electroportation.<br>As stated in the "Surface immunostaining of embryos" section of the Methods, we excluded large aggregates without DAPI or OCT4 stain. The rationale is that aggregates of unbound antibodies were occasionally retained inside the pre-amniotic cavity, due to the fixation step after surface staining.
Replication	All attempts at replication were successful. Each result presented is based on at least two and often more independent biological replicates, as indicated in Figure captions.
Randomization	For receptor mis-localization, breaking-junction and transwell experiments in vitro, human embryonic stem cells were randomly allocated to control and experimental groups.<br>For receptor mis-localization and visceral endoderm removal experiments, dissected embryos were randomly allocated to control and experimental groups.
Blinding	The investigators were not blinded.<br>Blinding was very challenging, if not impossible for our study. For example, mis-localized receptors show distinct cellular distribution, compared to controls, such as GFP and wild-type receptors. Second, breaking-junction leads to change of cell morphology and orientation. Third, transwell membrane produces unique background in fluorescent channel. Last, visceral endoderm removal results in significant decrease of embryo size.

Reporting for specific materials, systems and methods

We require information from authors about some types of materials, experimental systems and methods used in many studies. Here, indicate whether each material, system or method listed is relevant to your study. If you are not sure if a list item applies to your research, read the appropriate section before selecting a response.

Materials & experimental systems

n/a	Involved in the study	
[ ]	[x]	Antibodies
[ ]	[x]	Eukaryotic cell lines
[x]	[ ]	Palaeontology
[ ]	[x]	Animals and other organisms
[x]	[ ]	Human research participants
[x]	[ ]	Clinical data

Methods

n/a	Involved in the study	
[x]	[ ]	ChIP-seq
[x]	[ ]	Flow cytometry
[x]	[ ]	MRI-based neuroimaging

Antibodies

Antibodies used	As stated in the Antibodies section the Methods, we used:<br>BMPR1A (1:20, sc20736, Santa Cruz)<br>BRACHYURY/T (1:400, AF2085, R&D)<br>Clover (1:600, EMU101, Kerafast)<br>OCT4 (1:800, sc8628, Santa Cruz)<br>pSMAD1/5 (1:800, 13820s, Cell Signaling)<br>TGFBR1 (1:20, sc9048, Santa Cruz)<br>ZO-1 (1:100, 33-9100, Thermo Fisher)<br>ZO-1-FITC (1:100, 33-9111, Thermo Fisher)
Validation	BMPR1A (1:20, sc20736, Santa Cruz): validated by transient expression of tagged BMPR1A<br>BRACHYURY/T (1:400, AF2085, R&D): correctly stains mesoderm in E6.5 mouse embryos<br>Clover (1:600, EMU101, Kerafast): validated by transient expression of tagged BMPR1A-Clover<br>OCT4 (1:800, sc8628, Santa Cruz): validated by OCT4-RFP hESCs line. In addition, it correctly stain epiblast in E6.5 mouse embryos<br>pSMAD1/5 (1:800, 13820s, Cell Signaling): correctly stains mesoderm in E6.5 mouse embryos<br>TGFBR1 (1:20, sc9048, Santa Cruz): validated by transient expression of tagged TGFBR1<br>ZO-1 (1:100, 33-9100, Thermo Fisher): validated by ZO-1-GFP hESCs line. In addition, it correctly stain epiblast in E6.5 mouse embryos<br>ZO-1-FITC (1:100, 33-9111, Thermo Fisher): validated by ZO-1-GFP hESCs line. In addition, it correctly stain epiblast in E6.5 mouse embryos

Eukaryotic cell lines

Policy information about cell lines

Cell line source(s)	H1 cells (WiCell)
Authentication	Cells were cultured in media to maintain pluripotency and prevent differentiation. Plates showing evidence of differentiation were discarded. Cells were routinely stained for pluripotency markers to confirm stem cell identities.
Mycoplasma contamination	Cell lines were subject to routine PCR and biochemical mycoplasma tests and were negative for contamination.
Commonly misidentified lines (See ICLAC register)	H1 is not in the database

Animals and other organisms

Policy information about studies involving animals; ARRIVE guidelines recommended for reporting animal research

Laboratory animals	BL6 mouse
Wild animals	This study does not involve wild animals
Field-collected samples	This study does not involve field-collected samples
Ethics oversight	We have complied with all relevant ethical regulations for animal testing and research. Our use of animal is approved by Harvard University IACUC (protocol #15-01-229). Our use of human embryonic stem cells is approved by Harvard University IRB (protocol #IRB18-0665) and Harvard University ESCRO (protocol E00065).

Note that full information on the approval of the study protocol must also be provided in the manuscript.
